# Characteristics of Graphite Felt Electrodes Treated by Atmospheric Pressure Plasma Jets for an All-Vanadium Redox Flow Battery

**DOI:** 10.3390/ma14143847

**Published:** 2021-07-09

**Authors:** Tossaporn Jirabovornwisut, Bhupendra Singh, Apisada Chutimasakul, Jung-Hsien Chang, Jian-Zhang Chen, Amornchai Arpornwichanop, Yong-Song Chen

**Affiliations:** 1 Center of Excellence in Process and Energy Systems Engineering, Department of Chemical Engineering, Faculty of Engineering, Chulalongkorn University, Bangkok 10330, Thailand; tossaporn.jira@gmail.com (T.J.); justdao1994@gmail.com (A.C.); 2 Department of Mechanical Engineering and Advanced Institute of Manufacturing with High-Tech Innovations, National Chung Cheng University, Chiayi County 62102, Taiwan; bhup.bhu@gmail.com; 3 Graduate Institute of Applied Mechanics, National Taiwan University, Taipei 10617, Taiwan; R08543006@ntu.edu.tw (J.-H.C.); jchen@ntu.edu.tw (J.-Z.C.)

**Keywords:** all-vanadium redox flow battery, graphite felt, atmospheric pressure plasma jets, limiting current density, overpotential

## Abstract

In an all-vanadium redox flow battery (VRFB), redox reaction occurs on the fiber surface of the graphite felts. Therefore, the VRFB performance highly depends on the characteristics of the graphite felts. Although atmospheric pressure plasma jets (APPJs) have been applied for surface modification of graphite felt electrode in VRFBs for the enhancement of electrochemical reactivity, the influence of APPJ plasma reactivity and working temperature (by changing the flow rate) on the VRFB performance is still unknown. In this work, the performance of the graphite felts with different APPJ plasma reactivity and working temperatures, changed by varying the flow rates (the conditions are denoted as APPJ temperatures hereafter), was analyzed and compared with those treated with sulfuric acid. X-ray photoelectron spectroscopy (XPS) indicated that the APPJ treatment led to an increase in O-/N-containing functional groups on the GF surface to ~21.0% as compared to ~15.0% for untreated GF and 18.0% for H_2_SO_4_-treated GF. Scanning electron microscopy (SEM) indicated that the surface morphology of graphite felt electrodes was still smooth, and no visible changes were detected after oxidation in the sulfuric acid or after APPJ treatment. The polarization measurements indicated that the APPJ treatment increased the limiting current densities from 0.56 A·cm^−2^ for the GFs treated by H_2_SO_4_ to 0.64, 0.68, and 0.64 A·cm^−2^, respectively, for the GFs APPJ-treated at 450, 550, and 650 °C, as well as reduced the activation overpotential when compared with the H_2_SO_4_-treated electrode. The electrochemical charge/discharge measurements showed that the APPJ treatment temperature of 550 °C gave the highest energy efficiency of 83.5% as compared to 72.0% with the H_2_SO_4_ treatment.

## 1. Introduction

Renewable energy sources have attracted much attention due to the need for a reduction in CO_2_ emissions caused by the consumption of fossil fuels. Renewable energy sources, such as solar and wind power, are intermittent and fluctuate depending on the weather; as a result, there is a requirement of energy storage systems to stabilize the power output of renewable energy sources for robust energy management [1,2]. The energy storage system plays a key role in the penetration of renewable energy by providing smooth output, and it improves stability in the grid integration of renewable sources. Several types of energy storage systems have been developed to increase the energy capacity and roundtrip efficiency, as well as reduce the cost. Moreover, the self-discharge rate of the energy storage system is an important factor in large-scale energy storage, because a high self-discharge rate decreases the backup time of the energy storage system and the energy efficiency.

Among currently developed energy storage systems, VRFBs are considered as a preferred candidate to integrate with renewable energy sources. The VRFB was invented by Skyllas-Kazacos and coworkers in the 1980s [3]. In VRFBs, since both the positive and the negative electrolytes utilize the same metal ion at different oxidation states, the contamination due to crossover can be significantly decreased [3]. A special advantage of VRFBs is their flexible design in power rating and energy capacity. The power rating is determined by the stack size, including active area and cell number, while the energy capacity is influenced by the volume of the electrolyte reservoir and the concentration of the active species [4,5,6].

To enhance the performance of VRFBs, many research groups have been investigating their development in various fields such as the electrolyte solution, catalysts and additives, ion exchange membrane, flow field, electrode materials and their treatment, and performance and stability evaluation [5,6,7,8,9,10]. Since the electrochemical reaction takes place on the surface of the porous electrodes, the selection of electrode materials and their treatment plays a significant role in the enhancement of reaction kinetics of VRFBs. Currently, carbon papers (CPs), carbon felts (CFs), and graphite felts (GFs) have been widely employed as electrodes, due to their high porosity and permeability, high chemical stability, and high electrical conductivity [7,11,12].

The porous electrodes are in a compressed condition within the VRFB to reduce the contact resistance. The electrical, mechanical, and morphological properties of a porous electrode can be influenced by the compression ratio (CR) in a VRFB [13]. The optimal compression ratio of the porous electrode has been investigated in many studies [13,14,15,16]. Park et al. [14] showed that GFs with a compression ratio of 30% provided the maximum VRFB power and discharging capacity. However, GF with a CR of 20% presented the highest energy efficiency. In Wang’s study [15], the discharge time increased with increased CR, and the energy efficiency of a VRFB with a CR of 41.8% was improved by 19.4%. Ghimire et al. [16] showed that CF electrodes with a CR of 25% resulted in moderate pressure drops, low contact resistances, and uniform flow distribution. Hsieh et al. [17] experimentally investigated the effect of CR on the performance of a VRFB and demonstrated that the GFs compressed from 6.5 to 4 mm provided the optimal performance.

The inherent hydrophobic fiber surface retards the electrochemical reaction, and various surface modifications or treatments have been investigated for improvement of the redox reaction [4]. Zhou et al. [18] modified GF with ZrO_2_ for VRFBs and showed that ZrO_2_ nanoparticles improved the accessibility of electrolyte, as well as increased the activation sites for the redox reactions. As a result, both the electrochemical activity and the reversibility were enhanced. Hu et al. [19] reported a hybrid electrode comprising graphene oxide (GO), reduced graphene oxide, and graphene foam (GrF) to combine the high electrocatalytic activity of GO and high electrical conductivity of GrF. Pezeshki et al. [20] thermally treated the CP electrodes under various oxygen concentrations. They demonstrated that the activation overpotential was decreased by 100–140 mV in a 42% oxygen and 58% nitrogen atmosphere. Moreover, atmospheric pressure plasma jets (APPJs) for porous electrode treatment of VRFB were proposed in our previous study [21]. APPJs have already shown high potential in food processing, agriculture, rapid material processing, and surface modification with adhesion enhancement applications [22,23,24,25].

The use of atmospheric pressure plasma treatment for the enhancement of performance of various components of VRFBs has been reported in recent years [26,27,28]. Lin et al. [27] reported the performance enhancement of a VRFB by APPJ treatment of a carbon felt (CF) electrode. Recently, Chen et al. [28] reported the use of APPJ treatment on polydopamine-coated GF electrodes to alter the surface wettability and electrochemical activity for VRFB applications. Although APPJs have been shown to effectively improve the performance of VRFBs by increasing the oxygen-containing functional groups on the GF fiber surface, the effect of operating conditions of APPJs on the VRFB performance has not been widely studied. The synergetic effect of the temperature and reactive plasma species renders rapid material-processing capability. The plasma reactivity of the APPJ is related to the power input to the plasma and the decay process of the reactive species upon formation [29,30]. Increasing flow rate enhances the reactivity of plasma species but decreases the working temperature of the APPJ. 

Atmospheric pressure plasma systems have shown rapid material-processing capability due to the combined effect of the reactive plasma species and heat [31,32,33]. However, the efficacies of the surface modification of material caused by plasma reactive species and heat can most often be in opposite directions, especially in the case of carbonaceous materials, thus resulting in a tradeoff effect of plasma reactive species and heat on the surface modification, which needs to be investigated for the targeted APPJ application. Lin et al. [27] investigated, e.g., the effect of scan speed and time of APPJ treatment of the CF electrode, which showed that, although the oxygen-containing functional groups formed by plasma treatment increased the surface wettability, an over-etched surface led to decreased conductivity and electrochemical performance, indicating that the APPJ treatment conditions still need further investigation. In this regard, the tradeoff of the effects caused by plasma reactivity and heat was investigated via the modulation of flow rate in this study. Moreover, since acid treatment has been one of the convenient and widely used methods for improving the VRFB performance of carbonaceous electrodes [34,35,36,37,38], in this study, the H_2_SO_4_ treatment of GF electrodes was also performed in order to evaluate its performance compared to the APPJ treatment. In this study, the surface morphology, X-ray photoelectron spectroscopy (XPS), and Energy-dispersive spectroscopy (EDS) analyses of GF under acid treatment and APPJ treatment were compared. Moreover, to reduce the evaluation time of porous electrodes, the limiting current density of the VRFB with various treated electrodes was characterized as a performance index.

## 2. Materials and Methods

### 2.1. Preparation of a VRFB

**Figure 1:**
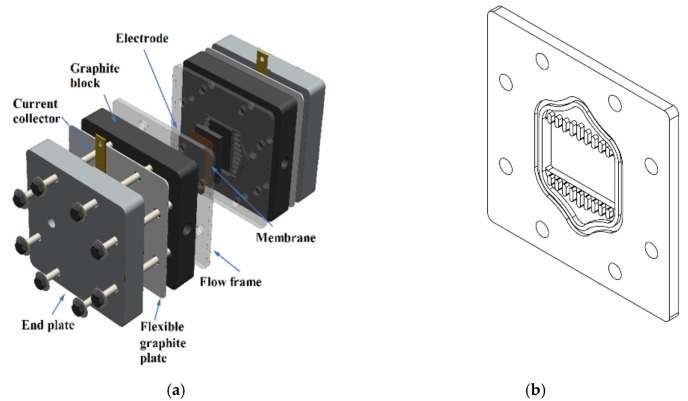
(**a**) Diagram showing different components of a VRFB assembly; (**b**) flow pattern on the PVC frame.

A single VRFB with an active area of 25 × 40 mm^2^ was designed for the performance measurement. The components of the VRFB, as shown in Figure 1a, include a proton exchange membrane (Nafion 117, Dupont, Wilmington, DE, USA), GF (GF650, CeTech, Taichung, Taiwan) porous electrodes, PVC frame, graphite plates, current collectors, and end plates. The PVC frame acts as a gasket to seal the cell, and flow patterns are designed on the PVC frame to uniformly distribute the electrolyte in the lateral direction, as shown in Figure 1b. The membrane was soaked in sulfuric acid at 60 °C for 24 h before use. A flexible graphite foil was placed between the current collector and the graphite plate to maximize the electrical contact. All components were sandwiched together by eight bolts and nuts with a torque of 4 N m.

For the acid treatment, the GFs with a dimension of 25 × 40 × 6.5 mm^3^ were soaked in 96% sulfuric acid for 12 h, followed by rinsing with deionized water. For the APPJ treatment, a scan-mode nitrogen DC-pulse APPJ (Industrial Technology Research Institute, Taiwan) was used to process the GF with various peak temperatures of ~450 °C, ~550 °C, and ~650 °C, by adjusting the nitrogen flow rates to 56, 40, and 30 standard liters per minute (SLPM), respectively. The schematic of the APPJ setup was described elsewhere [21]. The applied voltage, repetition frequency, and on/off duty cycle for the APPJ operation were 275 V, 25 kHz, and 7/33 μs, respectively. A quartz tube with a length of 37 mm and an inner diameter of 30 mm was installed downstream of APPJ to prevent the quenching effect from the ambient air, thereby increasing plasma jet length and reactivity. The gap between the quartz tube and the upper surface of the sample was 1 mm, to ensure a steady jet flow. The GFs were scanned twice with a scan rate of 0.75 cm/s.

**Figure 2:**
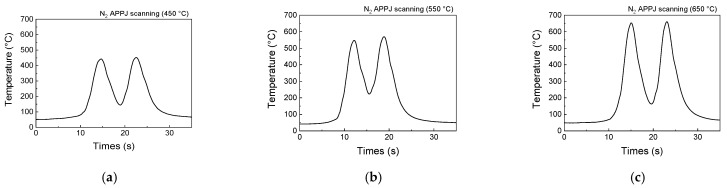
Temperature–time courses of GFs during APPJ treatments for nitrogen flow rates of (**a**) 56, (**b**) 40, and (**c**) 30 SLPM.

Figure 2 shows the temperature–time courses of GF surfaces with various nitrogen flow rates (monitored by a K-type thermocouple). The temperature increased rapidly when moving transversely under the plasma jet. The temperature increased when the GF was right under the plasma jet, and the temperature was low when graphite was away from the plasma jet. With the oscillation period of ~5–10 s, the temperature oscillated rapidly between 150 and 450 °C under an N_2_ flow rate of 56 SLPM, between 230 and 550 °C under an N_2_ flow rate of 40 SLPM, and between 90 and 650 °C under an N_2_ flow rate of 30 SLPM. The experimental conditions are denoted by the APPJ temperatures (APPJ 450 °C, 550 °C, and 650 °C) hereafter.

The electrolyte was prepared by dissolving 1.5 M VOSO_4_ in 2.0 M H_2_SO_4_ solution. Initially, the positive and negative electrolyte volumes were 100 and 50 mL, respectively. The electrolytes were recirculated between the VRFB and electrolyte tanks by using two diaphragm pumps (SMART digital DDA7.5-16AR-PVC/V/C, Grundfos, Denmark). The VRFB was pre-charged using a constant current of 0.04 A·cm^−2^ followed by a constant voltage of 1.71 V from the battery tester (PFX2021, Kikusui Electronic, Japan). During the pre-charge process, the V^4+^ was converted to V^5+^ in the positive side and to V^2+^ in the negative side. Then, half of the positive electrolyte was removed to make the electrolyte volumes in both reservoirs equal to 50 mL.

### 2.2. Experimental Procedure

For the VRFB equipped with GFs treated by different processes, the performance curves were measured by increasing current density with a step of 0.04 A·cm^−2^ from open-circuit voltage to a lower limit of 0.2 V. Then, the VRFB was charged and discharged between 0.7 and 1.71 V for 12 cycles. Data of the last 10 cycles were analyzed for coulombic efficiency (CE), voltage efficiency (VE), energy efficiency (EE), and discharging capacity (DC) using the following equations, respectively:

(1) $$CE=\frac{\int i_{\mathrm{discharge}}(t)dt}{\int i_{\mathrm{charge}}(t)dt},$$

(2) $$VE=\frac{V_{\mathrm{avg},\mathrm{discharge}}}{V_{\mathrm{avg},\mathrm{charge}}},$$

(3) EE=CE×VE,

(4) $$DC=\int i_{\mathrm{discharge}}(t)dt.$$

After the cyclic charge/discharge processes, the performance curve of the VRFB was measured again to evaluate the variation in limiting current density. Moreover, GF electrodes were examined by X-ray photoelectron spectroscopy (XPS, Sigma Probe, Thermo VG Scientific, Waltham, MA, USA) with a monochromated Al K_α_ X-ray source and energy-dispersive spectroscopy (EDS). The surface morphology of GF fibers was observed by field-emission scanning electron microscopy (FE-SEM, S-4800 Hitachi, Hitachi High-tech Corp., Tokyo, Japan).

## 3. Results and Discussion

### 3.1. Characteristics of GF Electrodes

**Figure 3:**
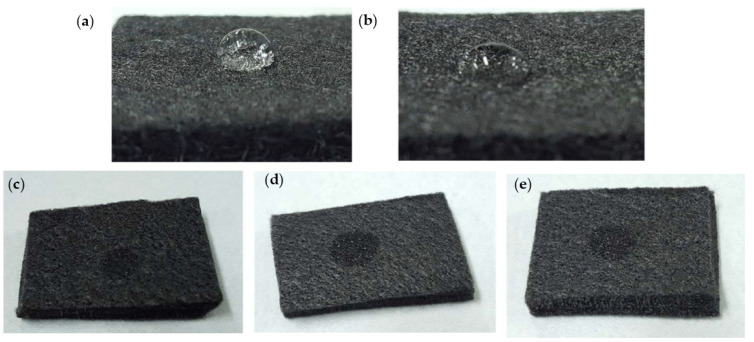
Photographic images indicative of the wettability of GF electrode: (**a**) untreated; (**b**) H_2_SO_4_ treatment; (**c**) APPJ 450 °C treatment; (**d**) APPJ 550 °C treatment; (**e**) APPJ 650 °C treatment.

Figure 3 shows the photographic images indicative of the wettability of GF with various treatments. According to the general visualization of these images, it can be inferred that the contact angle of the water droplet on the surface of untreated GF (Figure 3a) was larger than that of H_2_SO_4_-treated GF (Figure 3b). Sulfuric acid helps improve the hydrophilicity of the GF fiber surface. The hydrophilicity of the GF can be further enhanced by APPJ treatment. As can be seen for APPJ-treated specimens in Figure 3c–e, water droplets were completely absorbed into the GF electrodes. However, the wettability level could not be differentiated by water droplets; therefore, further investigation on the characteristics of GF is required.

**Figure 4:**
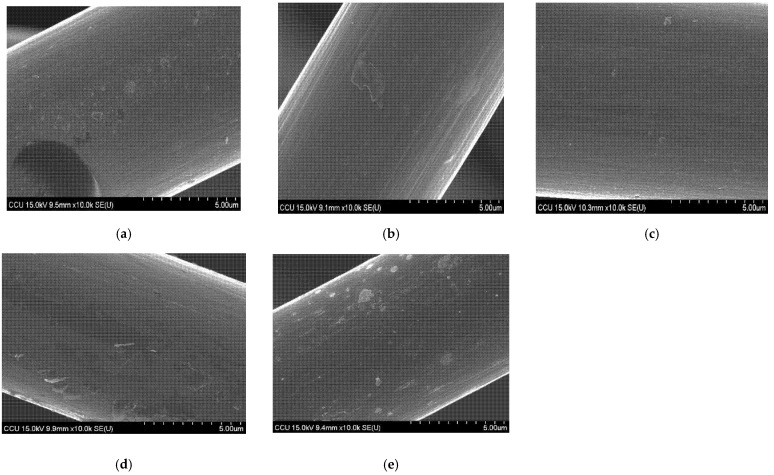
SEM images of GF electrode: (**a**) untreated; (**b**) H_2_SO_4_ treatment; (**c**) APPJ 450 °C treatment; (**d**) APPJ 550 °C treatment; (**e**) APPJ 650 °C treatment.

Figure 4 shows the surface morphology of untreated treated and treated GFs. It can be seen, in Figure 4a, that some impurities existed on the surface of the untreated GF fibers. After the treatment by H_2_SO_4_ solution, some impurities vanished and the fiber surface became smoother; however, some impurities remained on the fiber surface, as shown in Figure 4b. After the treatment by APPJ at 450 °C, the fiber became rougher with some visible cavities, as shown in Figure 4c, serving as active sites for the redox reaction of the electroactive vanadium species. When the APPJ treatment temperature increased to 550 °C and 650 °C, the GF fiber surface became much rougher and parts of the fiber surface were peeled off, as shown in Figure 4d,e. The peeled fiber surface could contribute to the improvement of wettability, resulting in the enhancement of electrochemical reaction on the fiber surface.

**Figure 5:**
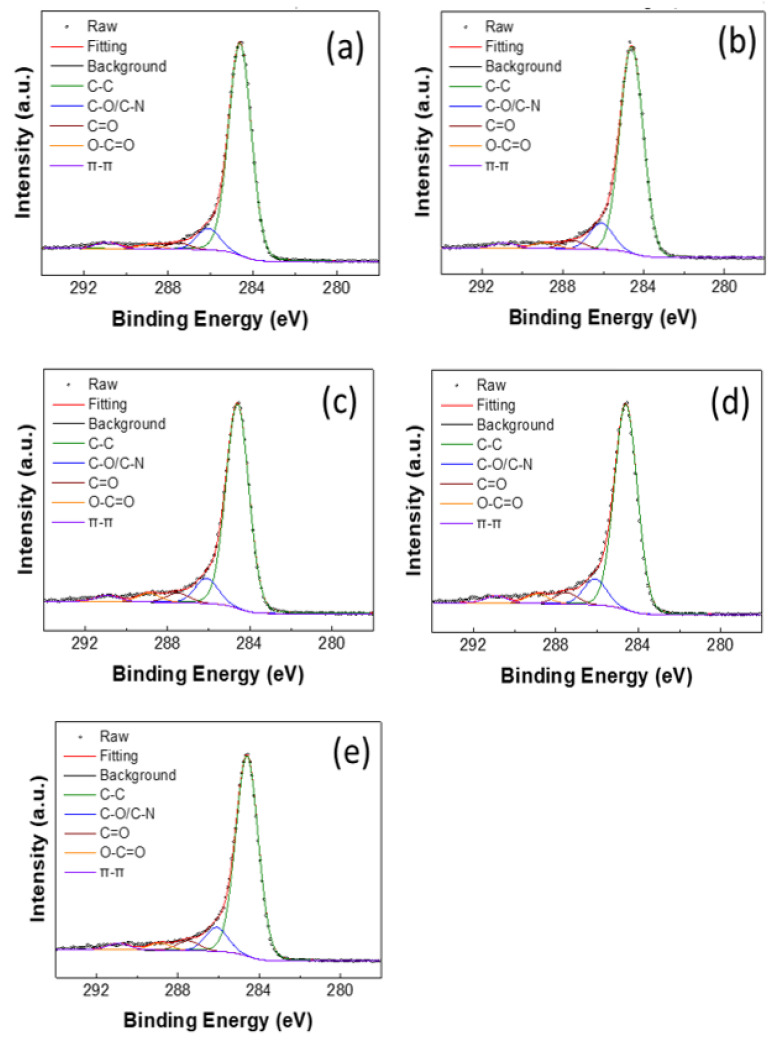
XPS images of GF electrode: (**a**) untreated; (**b**) H_2_SO_4_ treatment; (**c**) APPJ 450 °C treatment; (**d**) APPJ 550 °C treatment; (**e**) APPJ 650 °C treatment.

In addition to the morphological study of different GFs, the surface chemistry of GFs was also revealed by XPS. In the present study, the peak fitting of C 1*s* was carried out and data are shown in Figure 5. Using the deconvoluted C 1*s* peak, the relative concentrations of different functional groups were calculated, as summarized in Table 1. As can be seen in Figure 5a, the untreated GF exhibited a major peak at 284.6 eV for C–C bonding (84.8%), as well as minor peaks at 286.1, 287.5, and 289.9 eV for C–O (8.6%), C=O (2.5%), and O–C=O (1.7%), respectively. Additionally, a shakeup peak for π–π* transition (2.4%) was observed at 290.9 eV [34]. When the GF was treated with H_2_SO_4_, a significant decrease in the C–C bonding content (82.0%) was observed. The effect of sulfuric acid treatment on the oxygenated carbon in graphite has been shown to increase the number of oxygenated groups; however, when it comes to the relative contents of various oxygenated carbon groups, diverse observations have been reported in XPS analysis [34,35,36,37,38]. Sun et al. [35] reported that, after H_2_SO_4_ treatment, both the C–O and C=O contents increased dramatically. Similarly, Duman and Ficicilar [36] reported a decrease in C=C content and an increase in C–C, C–O, and C=O contents after acid treatment. On the other hand, Gao et al. [37] reported that, after H_2_SO_4_ treatment, the C–O content increased, whereas C–C and C=O contents decreased. Similarly, Li et al. [38] reported that C–C and C–O contents decreased while C=O and O–C=O contents increased after acid treatment. In the present study, it can be seen from Table 1 that the acid treatment has reduced the percentage of C–C (82.0%) and π–π* transition (2.1%) but increased the percentage of C–O (10.4%), C=O (3.2%), and O–C=O (2.3%). The accumulative percentage of all oxygenated groups was 12.8% in the untreated GF, but increased to 15.9% in the acid-treated GF. The π–π* transition component of C 1*s* XPS spectra is associated with the *sp*^2^ hybridized carbon, and the ratio of the contribution of π–π* transition to that of C–C bonding can be an indication of the *sp*^2^/*sp*^3^ carbon ratio. This ratio for untreated GF was 0.028 but decreased to 0.025 for acid-treated GF. The lower *sp*^2^/*sp*^3^ carbon ratio can be an indication of decreased graphitic nature of the surface in the acid-treated GF and may have implications for the conductivity [39,40].

The N_2_ APPJ treatment of GF led to the introduction of oxygen-containing functional groups and nitrogen doping at the surface [21]. In the present study, when GF was treated with APPJ at 450 °C, the XPS data, compared to the untreated GF, showed a decrease in the percentage of C–C (80.5%) but an increase in the percentage of C–O/C–N (9.9%), C=O (3.7%), and O–C=O (3.4%). Additionally, the accumulative percentage of overall oxygen/nitrogen-containing carbon groups increased to 17.0%. Similarly, when GF was treated with APPJ at 550 °C, the C–C contribution decreased to 79.1% but the contributions of C–O/C–N, C=O, and O–C=O were increased to 10.0%, 4.4%, and 3.7%, respectively. Moreover, the accumulative percentage of overall oxygen/nitrogen-containing carbon groups increased to the maximum of 18.1%. However, for the APPJ treatment at 650 °C, the accumulative percentage of overall oxygen/nitrogen-containing carbon groups decreased to 16.6% with the individual contributions of C–O/C–N, C=O, and O–C=O groups at 9.7%, 4.0%, and 2.9%, respectively. Therefore, it is clear from the XPS data that the APPJ treatment led to the improved oxygenation of the GF surface, with the APPJ treatment at 550 °C being the most effective. Moreover, the increase in surface wettability of APPJ-treated GFs (Figure 3c–e) can be attributed to the improved surface oxygenation. However, it is important to mention that, although the acid-treated GF and the GF APPJ-treated at 650 °C had comparatively the same level of oxygenated groups (15.9% and 16.6%, respectively), the level of visible surface wettability was not the same, as shown in Figure 3b,e. Nevertheless, the ratio of the contribution of π–π* transition to that of C–C bonding for the APPJ-treated GF was 0.03, 0.035, and 0.031, respectively, in order of increasing temperature, and this can be an indication of increased graphitic nature of the surface in the APPJ-treated GFs [40].

### 3.2. Effect of Treatment Methods on Efficiency and Discharge Capacity

**Figure 6:**
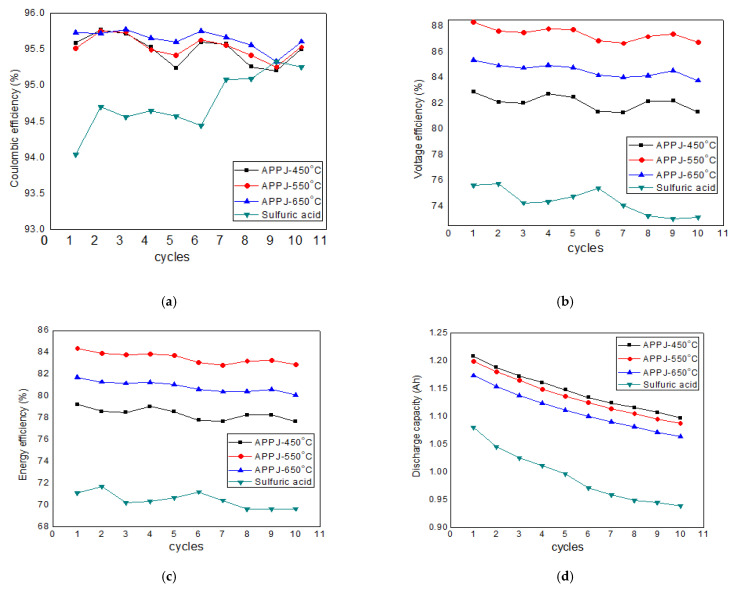
Performance of the VRFB with various GFs: (**a**) coulombic efficiency; (**b**) voltage efficiency; (**c**) energy efficiency; (**d**) discharge capacity.

The VRFB with GFs treated by various methods was operated for 12 cycles, and the data from the last 10 cycles were used to evaluate the performance. Figure 6 shows the performance of the VRFB with various GFs. Figure 6a indicates that the coulombic efficiencies varied between 94% and 95.5%. The CE for H_2_SO_4_-treated GF was ~94.0% which increased to ~95.5% for the APPJ-treated GFs; however, the variations in the APPJ-treatment temperature showed no significant effect on the coulombic efficiency. Figure 6b shows that voltage efficiencies of VRFBs with APPJ-treated GFs were between 82% and 88%, whereas it was between 73% and 76% for VRFB with H_2_SO_4_-treated GF electrode. The exact reason for the lower CE and VE of the H_2_SO_4_-treated GF, compared to the APPJ-treated GF, is not known, but we speculate it may have been due to the voltage loss arising from the increased ohmic polarization [41] originating from the lower graphitic nature of the acid-treated GF surface, as mentioned previously on the basis of the XPS data. The energy efficiency for H_2_SO_4_-treated GF was ~72.0% which increased to ~83.5% for the APPJ-treated GFs. As evident from Equation (3), EE is the product of CE and VE. Since the difference in the CEs of the VRFBs with these GFs was not noticeable, the energy efficiency was dominated by the voltage efficiency. As a result, the variation in energy efficiency was similar to that of the voltage efficiency, as shown in Figure 6c. Moreover, the VRFB performance of plasma-treated carbonaceous electrodes is summarized in Table 2. As can be seen, the energy efficiency of the present APPJ-treated GF was comparable to that of the APPJ-treated CF electrodes [27] and GF electrodes [28], but it was lower than that of the low-pressure plasma-treated graphene-incorporated GF electrodes recently reported by Bellani et al. [42], which could be attributed to the additional contribution of incorporated graphene toward an expected enhancement in catalytic and electronic conductivity. Figure 6d shows that the discharge capacity slightly decreased with the cycling number. This could have been due to imperfectly sealed electrolyte tanks, resulting in gradual oxidation of the electrolyte. The possible reasons are discussed in the next section. Moreover, the air oxidation of V^2+^ in the negative half-cell led to a reduction in battery performance due to the limiting of active species in the negative electrolyte [43].

### 3.3. Effect of Treatment Methods on Limiting Current Density

**Figure 7:**
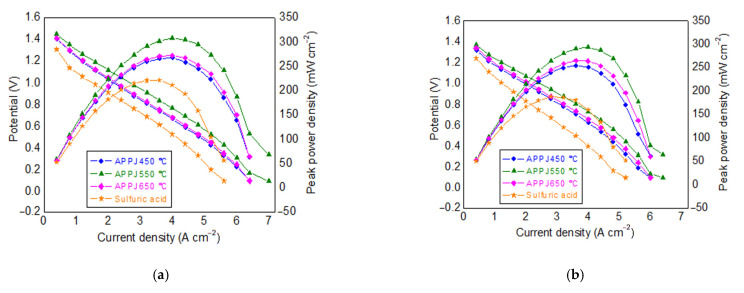
Performance curves of VRFB with GFs treated by various methods: (**a**) before cycling test; (**b**) after cycling test.

The limiting current density of the VRFB was obtained by estimating the intercepts of polarization curves on the horizontal axis, where the cell voltage is zero. The polarization curves of the VRFB with various GFs before and after the cycling tests are shown in Figure 7a,b, respectively. As can be seen, the limiting current densities for GFs treated by H_2_SO_4_, APPJ 450 °C, APPJ 550 °C, and APPJ 650 °C were 0.56, 0.64, 0.68, and 0.64 A·cm^−2^, respectively. The GF treated by APPJ 550 °C showed both the maximum limiting current density and the maximum energy efficiency, followed by APPJ 450 °C, APPJ 650 °C, and lastly H_2_SO_4_-treated GF. As a result, the energy efficiency was closely correlated with the limiting current density obtained from polarization curves. In addition, the key operating conditions, current density, and electrolyte flow rate also affected the battery performance with their own characteristics. The high current density caused an increase in overpotentials, but it also gave a high-power output, whereas an increase in electrolyte flow could decrease the concentration overpotential but consume more energy for supplying the pumps.

In Figure 7a,b, the initial voltage obtained for H_2_SO_4_-treated GF was ~0.2 V lower as compared to the APPJ-treated GFs. In polarization measurements, the voltage drop at low current densities is primarily attributed to the activation overpotentials. The higher voltage in the case of APPJ-treated GFs could, therefore, indicate that APPJ treatment is more favorable, compared to the H_2_SO_4_-treatment, for enhancing the electrocatalytic activity of GF electrodes. Moreover, for APPJ-treated GFs, the voltage obtained with the APPJ 550 °C was slightly higher than that with the APPJ 450 °C. However, the obtained voltage dropped to lower values when the treatment temperature was further increased to 650 °C (for APPJ 650 °C). Lin et al. [27] observed that, for the CF electrodes treated with APPJ multiple times, although the APPJ treatment enhanced the hydrophilicity, it decreased the electronic conductivity of the felt. In the present study, although no electronic conductivity measurement was performed, the changes in the overall graphitic nature of various APPJ-treated GFs, as previously discussed based on the XPS data in Section 3.1, can be considered as an indicative of the potential electronic conductivity of the APPJ-treated GFs, where the graphitic nature of the GF increased upon increasing the APPJ treatment temperature from 450 °C to 550 °C but then decreased for AAPJ 650 °C. Nevertheless, this indicates that the APPJ treatment temperature may have a tradeoff effect between surface functionalization and wettability, as well as between surface degradation and electronic conductivity of the GF electrodes [27,45]; therefore, careful considerations must be kept in mind in this regard. 

## 4. Conclusions

The GF electrodes for an all-vanadium redox flow battery (VRFB) were developed by the sulfuric acid treatment and by APPJ treatment. To identify the optimal conditions for the APPJ treatment, the performance of the various GF electrodes APPJ-treated in the 450–650 °C temperature range (adjusted by varying flow rates) was evaluated and compared with the performance of the sulfuric acid-treated GF electrode. The APPJ treatment increased the population of O-/N-containing functional groups to ~21.0% as compared to ~15.0% for untreated GF and 18.0% for H_2_SO_4_-treated GF. However, no discernable variations were detected in terms of the surface morphology after H_2_SO_4_ treatment or APPJ treatment. Moreover, the electrochemical result shows that the electrode treated with 550 °C APPJ gave the highest battery efficiency, as well as increased the limiting current density and voltage efficiency, due to the presence of a high number of oxygen-containing groups at the surface. The CE, VE, and EE of VRFBs with 550 °C APPJ-treated GF were 95.5%, 88.0%, and 83.5% at a current density of 0.04 A·cm^−2^, which was higher than the acid-treated GF electrodes and comparable with the plasma-treated carbonaceous electrodes. It is clear that the electrode treatment with APPJs significantly increased the battery performance compared to the sulfuric acid treatment, due to the high wettability and the surface oxygen functional groups. However, APPJ treatment at high temperature may cause the degradation of electrode fiber, which can lead to a reduction in electrochemical activity and voltage efficiency.

## Figures and Tables

**Table 1:** Relative percentage of different groups in C 1*s* spectra.

AtomicRatio (%)	C–C	C–O/C–N	C=O	–COOH	π–π
As deposited	84.8	8.6	2.5	1.7	2.4
H_2_SO_4_-treated	82.0	10.4	3.2	2.3	2.1
APPJ 450 °C	80.5	9.9	3.7	3.4	2.4
APPJ 550 °C	79.1	10	4.4	3.7	2.8
APPJ 650 °C	80.9	9.7	4.0	2.9	2.5

**Table 2:** A comparison of some plasma-treated carbonaceous electrodes with the present work. The quantity in bracket in the first column indicates the values of constant current density, in A·cm^−2^_,_ during the charge/discharge process.

Electrode Material with Treatment Method	CE (%)	VE (%)	EE (%)	Ref.
H_2_SO_4_-treated GF (0.04)	~94.5	~76.0	72.0	This work
APPJ 450 °C-treated GF (0.04)	~95.5	~82.0	79.0	This work
APPJ 550 °C-treated GF (0.04)	~95.5	~88.0	83.5	This work
APPJ 650 °C-treated GF (0.04)	~95.5	~85.0	81.0	This work
APPJ-treated polydopamine coated GF (0.04)	85.2	93.8	79.9	[28]
APPJ-treated CF (jet speed 5 mm·s^−1^; single scan) (0.12)	–	–	~84.2	[27]
APPJ-treated CF (jet speed 5 mm·s^−1^; single scan) (0.14)	–	–	~82.8	[27]
APPJ-treated CF (jet speed 5 mm·s^−1^; single scan) (0.16)	97.0	79.9	~77.6	[27]
APPJ-treated CF (jet speed 10 mm·s^−1^; single scan) (0.12)	–	–	~80.0	[27]
APPJ-treated CF (jet speed 5 mm·s^−1^; double scan) (0.14)	–	–	~81.7	[27]
APPJ-treated CF (jet speed 5 mm·s^−1^; triple scan) (0.14)	–	–	~81.9	[27]
Low-pressure plasma-treated graphene-incorporated GF (0.05)	~97.0	~92.5	90.8	[42]
Low-pressure plasma-treated graphene-incorporated GF (0.80)	~98.0	<70.0	<75.0	[44]
Scan mode APPJ-treated GF (0.04)	92.5	88.0	81.4	[21]
Spot mode APPJ-treated GF (0.04)	92.4	89.4	82.7	[21]

## Data Availability

The data presented in this study are available on reasonable request from the corresponding author.
